# Michaelis–Menten kinetics during dry etching processes

**DOI:** 10.1371/journal.pone.0299039

**Published:** 2024-03-01

**Authors:** Rimantas Knizikevičius

**Affiliations:** Department of Physics, Kaunas University of Technology, Kaunas, Lithuania; Federal University of ABC, BRAZIL

## Abstract

The chemical etching of germanium in Br_2_ environment at elevated temperatures is described by the Michaelis–Menten equation. The validity limit of Michaelis–Menten kinetics is subjected to the detailed analysis. The steady-state etching rate requires synergy of two different process parameters. High purity gas should be directed to the substrate on which intermediate reaction product does not accumulate. Theoretical calculations indicate that maximum etching rate is maintained when 99.89% of the germanium surface is covered by the reaction product, and 99.9999967% of the incident Br_2_ molecules are reflected from the substrate surface. Under these conditions, single GeBr_2_ molecule is formed after 30 million collisions of Br_2_ molecules with the germanium surface.

## 1. Introduction

Michaelis–Menten equation describes relationship between the formation rate of single reaction product and the concentration of single reactant. The Michaelis–Menten saturation curves are similar to the etching rate dependences on the concentration of reactive species. The similarity is usually observed during dry etching of elemental substrates [1]. The removal of uppermost monolayer exposes another monolayer of the same substrate. During the etching process, the adsorbed layer is constantly replenished by the reaction product, which eventually desorbs [2]. Although, the etching rate is measured in the monolayers per second, the substrate surface can be considered unchanged [3]. In some cases, the etching process results in the evolution of the surface morphology, especially when the substrate surface is contaminated by the unreactive compounds [4, 5].

Dry etching processes occur at the atomic scale. Therefore, establishing relationship between dry etching processes and Michaelis–Menten kinetics is beneficial. The obtained theoretical results can be used to optimize synthesis of nanozymes. Despite that Michaelis–Menten equation introduces two compatibility issues on the dry etching processes:

1. single reactant must be used. The number of artefacts significantly increases during plasma etching processes [6]. The working pressure in the typical ICP reactor varies from 1 to 100 mTorr [7], and the measurement of absolute concentrations of reactive species in the plasma requires special design of the experimental system [8, 9]. Therefore, molecular reactants are preferred;
2. the elemental substrates must be used. Compounds made of two or more chemical elements are not suitable because mathematical description of the dry etching processes results in too complex etching-rate expressions [10, 11].

After literature review, the experiment [12] is selected for establishing relationship between dry etching processes and Michaelis–Menten kinetics. The selection is based on the following criteria:

1. the partial pressure of Br_2_ molecules is varied from 0 to 200 Torr. This allows to test the limits of dry etching processes, predicted by the Michaelis–Menten equation;
2. the etching rate in the saturation regime is measured over temperature range (453 ÷ 626) *K*. This allows to check the dependence of Michaelis constant on temperature;
3. the measurements consist of the large number of datapoints. This allows to perform the reliable statistical analysis;
4. theoretical analysis of the experimentally measured germanium etching rate dependences on the partial pressure of Br_2_ molecules yields the activation energies of elementary processes.

The description of experimental setup is presented in the separate section in order to provide more complete view of the etching process. The information is sourced from work [12] and references therein as well as associated publications by the same authors.

## 2. Experimental

The chemical etching of Ge substrates was performed in the isothermal gas-flow reactor using Br_2_+Ar mixture. Before the etching process the reagent-grade liquid bromine was purified in the distillation process. While, Ar gas was purified using Ni-Cr catalyst and zeolite adsorbates in order to remove oxygen and water vapor. The concentration of Br_2_ molecules was measured by gas-phase titration with molecular iodine. During the experiment, gallium-doped Ge(111) substrates with electrical resistivity 4.8 Ω cm were used. Prior to the etching process germanium substrates were cleaned. During cleaning procedure the substrates were initially ground mechanically with corundum powder, and subsequently carefully rinsed with deionized water. Later, the substrates were immersed for 8 minutes in the following mixture of aqueous solutions: 2 ml (10% NaOH) and 100 ml (30% H_2_O_2_). The chemical etching rate of germanium substrates was measured using semi-microbalances.

## 3. Theory

At standard conditions, germanium dibromide is white crystalline solid. Every GeBr_2_ molecule has 2 dangling bonds and can form chemical bonds with the adjacent molecules [13]. The monoclinic crystals start to melt at temperature about 395 K. Therefore, chemical etching of germanium in Br_2_ environment is possible only at elevated temperatures. On the other hand, germanium substrates start to melt at temperature 1211 K. In the considered temperature range, Br_2_ molecules from the gas phase chemisorb on the substrate surface and subsequently form GeBr_2_ molecules:

$$Ge\left(s\right)+Br_{2}\left(g\right)\to GeBr_{2}\left(a\right).$$ (1)

According to the transition state theory (TST), which is described in work [14], the reaction rate constant is equal to

$$k_{r}=A\nu_{TST}\exp\left(\frac{\Delta S}{k}\right)\exp\left(-\frac{\Delta H}{kT}\right),$$ (2)

where A is the average kinetic transmission coefficient, v_TST_ = kT/h is the lattice atom oscillation frequency, h is the Planck constant, k is the Boltzmann constant, T is the temperature, ΔS is the activation entropy, and ΔH is the activation enthalpy. The reaction activation energy E_r_ linearly depends on the activation enthalpy [15]. These two physical quantities differ little, and the activation enthalpy is usually assumed to be equal to the reaction activation energy. The activation entropy is negligible because the reaction, defined by Eq. (1), occurs only at elevated temperatures. As the result, the reaction rate constant takes the following form:

$$k_{r}=A\nu_{TST}\exp\left(-E_{r}/kT\right).$$ (3)

When the etching rate is measured accurately, the maximum absolute error of the reaction activation energy is equal to

$$\Delta E_{r}=\frac{\Delta k_{r}}{k_{r}}E_{r}.$$ (4)

GeBr_2_ molecules form the adsorbed layer of one-monolayer thickness [16, 17]. Their relative concentration in the adsorbed layer is equal to

$$c=\left[GeBr_{2}\right]/C,$$ (5)

where C = 7.29 × 10^14^ cm^-2^ is the planar density of Ge(111) substrates. GeBr_2_ molecules diffuse in the adsorbed layer until eventually desorb

$$GeBr_{2}\left(a\right)\to GeBr_{2}\left(g\right).$$ (6)

The desorption process is characterized by the desorption rate constant

$$\omega =\nu_{TST}\exp\left(-E_{d}/kT\right),$$ (7)

where E_d_ is the desorption activation energy. When the etching rate is measured accurately, the maximum absolute error of the desorption activation energy is equal to

$$\Delta E_{d}=\frac{\Delta \omega }{\omega }E_{d}.$$ (8)

The following differential equation includes earlier mentioned elementary processes and describes the concentration kinetics in the adsorbed layer:

$$\frac{dc}{dt}=\beta k_{r}p-\omega c,$$ (9)

where β = 1-Θ is the surface fraction not covered with adsorbate, Θ = c is the surface coverage, p is the partial pressure of Br_2_ molecules, and t is the etching time. The concentration of GeBr_2_ molecules in the adsorbed layer at steady-state regime is equal to

$$c_{st}=\frac{k_{r}p}{k_{r}p+\omega }.$$ (10)

The etching rate is equal to the desorption rate of GeBr_2_ molecules

$$V=\frac{k_{r}p\;\omega }{k_{r}p+\omega }.$$ (11)

According to the L’Hôpital’s rule, the etching rate at extremely high pressure reaches maximum value

$$V_{\max}=\omega .$$ (12)

The normalized etching rate at steady-state regime is equal to

$$\frac{V}{V_{\max}}=\frac{\;1}{1+\omega /\left(k_{r}p\right)}.$$ (13)

It is important to note that the chemical etching rate of germanium is described by the section of right rectangular hyperbola. This enables to describe the chemical etching rate of germanium using the Michaelis–Menten equation

$$V=\frac{V_{\max}p\;}{K_{M}+p},$$ (14)

where K_M_ = ω/k_r_ is the Michaelis constant, which is equal to the partial pressure at which the etching rate reaches half of its maximum value. The etching rate is calculated in monolayers per second. The monolayer thickness is evaluated using the following equation:

$$h_{0}=\sqrt[{3}]{\frac{M_{Ge}}{\rho_{Ge}N_{A}}},$$ (15)

where ρ_Ge_ is the density of germanium, M_Ge_ is the molar mass of germanium, and N_A_ is the Avogadro constant. In the experiment [12], the etching rate was measured in g-atom*cm^-2^s^-1^. The chemical etching rate of germanium is converted into nm/min using the following equation:

$$V=\frac{\Delta m_{S}}{\rho_{Ge}St},$$ (16)

where Δm_s_ is the mass loss of Ge substrate and S is the substrate surface area.

## 4. Results and discussion

**Fig 1:**
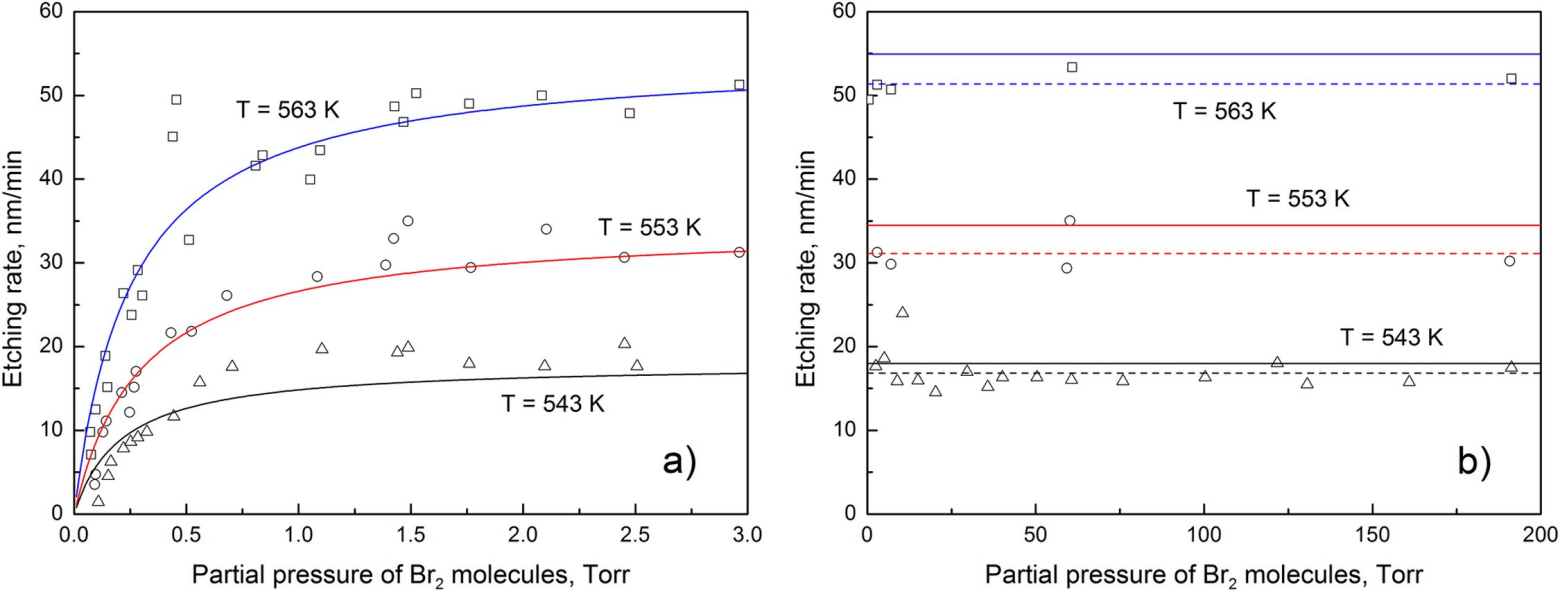
The experimental [12] and theoretical [18] dependences of germanium etching rate on the partial pressure of Br_2_ molecules at three different temperatures. The fitting is performed using the Origin Pro software.

The chemical etching of germanium in Br_2_ environment is investigated using the nonlinear regression of the experimental data. The experimental and theoretical dependences of germanium etching rate on the partial pressure of Br_2_ molecules at different temperatures are shown in Fig 1. It is observed that chemical etching rate increases with the increase in temperature. The nonlinear regression of the experimental data provides reasonable fits at low partial pressure. However, the difference between experimental and theoretical dependences becomes pronounced at high partial pressure. The statistical software struggles to provide accurate values of the desorption rate constants because of the scattered experimental data points at low partial pressure of Br_2_ molecules. According to Eq. (8), the desorption activation energy is also affected by the fitting errors. In order to address the discrepancy, graphical analysis of the experimental data is performed. The Michaelis–Menten saturation curves at high partial pressure are presented in Fig 1B by the dashed lines. During the calculations of uncertainties, the absolute error of the desorption rate constant is assumed to be equal to the standard deviation from the average etching rate. The kinetic parameters, determined during the nonlinear regression and graphical analysis of the experimental data, are presented in Table 1. The reaction rate constants are derived numerically because graphical analysis methods are inaccurate at low partial pressure. It is found that the activation energy of Ge(s)+Br_2_(g) → GeBr_2_(a) reaction is equal to (1.168 ± 0.173) eV. Graphical analysis of the experimental data yields lower values of the desorption rate constants. However, the influence of analysis method on the desorption activation energy of GeBr_2_ molecules is very small. The nonlinear regression analysis with fixed ω is also performed in order to evaluate the influence of fitting errors on the reaction rate constants. It is found that the reaction rate constants are at least 1.5 times more sensitive to the considered fitting errors than the desorption rate constants. This statistical finding provides additional evidence that the reaction activation energy is lower than the desorption activation energy.

**Table 1:** The kinetic parameters determined during nonlinear regression and graphical analysis of the experimental data. Activation energies of the elementary processes are calculated using TST. The average kinetic transmission coefficient A = 1pa^-1^.

Temperature, K	k_r_±Δk_r_,Torr^-1^s^-1^	E_r_±ΔE_r_,eV	ω±Δω,s^-1^	E_d_±ΔE_d_,eV
Nonlinear regression analysis				
543	4.975±1.045	1.166±0.245	1.060±0.038	1.404±0.050
553	6.859±0.839	1.173±0.143	2.032±0.070	1.399±0.048
563	12.670±1.433	1.164±0.132	3.237±0.107	1.403±0.046
Michaelis–Menten saturation curve				
543			0.992±0.125	1.408±0.177
553			1.834±0.134	1.404±0.103
563			3.025±0.085	1.406±0.040
Nonlinear regression analysis with fixed ω				
543	5.558±1.232	1.161±0.257	0.992	1.408
553	8.072±1.110	1.165±0.160	1.834	1.404
563	14.380±1.523	1.158±0.123	3.025	1.406

Desorption activation energy of GeBr_2_ molecules defines the chemical etching rate of germanium in the saturation regime. In the work [12], desorption activation energy of the reaction product was derived graphically. However, the Arrhenius plot yielded single approximate value of the lattice atom oscillation frequency 1.37×10^13±1^s^-1^ in the temperature range (453 ÷ 626)K. Let us investigate the saturation regime in the Michaelis–Menten saturation curves using TST, which enables to calculate the lattice oscillation frequency as well as the desorption activation energy for every data point. The theoretical results obtained from the reanalysed experimental data are presented in Table 2. According to TST, the lattice oscillation frequency in the considered temperature range varies from 9.445×10^12^ to 1.305×10^13^s^-1^, and the average desorption activation energy of GeBr_2_ molecules is equal to (1.397±0.014)eV. The absolute error of the desorption activation energy is assumed to be equal to the standard deviation from the average desorption activation energy. The desorption activation energy of the reaction product, derived in the experiment [12], is equal to (1.430±0.043)eV. However, the authors wrongly assumed that the derived value corresponds to the desorption activation energy of GeBr_4_ molecules. It is important to note that the Arrhenius plot does not provide any information about the chemical formula of the reaction product. Despite the mistake made in work [12], identifying prevailing reaction product, the desorption activation energies are very similar. The usage of TST reduced uncertainty in the desorption activation energy more than three times.

**Table 2:** The kinetic parameters derived from etching rates in the saturation regime.

Temperature, K	$V_{\max},\;\frac{g-atom}{cm^{2}s}$	$V_{\max},\;\frac{nm}{\min}$	ω, s^-1^	ν_TST_, s^-1^	E_d_, eV
453	2.933×10^−12^	0.02401	0.001414	9.445×10^+12^	1.423
453	3.861×10^−12^	0.03161	0.001862	9.445×10^+12^	1.413
450	4.300×10^−12^	0.03520	0.002073	9.376×10^+12^	1.398
458	7.542×10^−12^	0.06174	0.003637	9.551×10^+12^	1.402
463	8.810×10^−12^	0.07212	0.004248	9.654×10^+12^	1.412
448	1.041×10^−11^	0.08527	0.005022	9.342×10^+12^	1.358
464	1.323×10^−11^	0.1083	0.006379	9.667×10^+12^	1.398
476	2.523×10^−11^	0.2065	0.01217	9.910×10^+12^	1.407
476	3.611×10^−11^	0.2956	0.01741	9.920×10^+12^	1.394
521	6.518×10^−10^	5.336	0.3143	1.087×10^+13^	1.401
526	1.002×10^−9^	8.206	0.4834	1.096×10^+13^	1.394
544	2.578×10^−9^	21.10	1.243	1.134×10^+13^	1.399
548	3.646×10^−9^	29.85	1.758	1.142×10^+13^	1.394
558	5.095×10^−9^	41.71	2.457	1.163×10^+13^	1.404
562	7.381×10^−9^	60.43	3.559	1.170×10^+13^	1.395
563	1.019×10^−8^	83.45	4.915	1.172×10^+13^	1.382
589	3.534×10^−8^	289.3	17.04	1.228×10^+13^	1.386
605	6.503×10^−8^	532.4	31.36	1.260×10^+13^	1.392
619	1.012×10^−7^	828.5	48.80	1.290×10^+13^	1.403
626	2.049×10^−7^	1677	98.80	1.305×10^+13^	1.382

**Fig 2:**
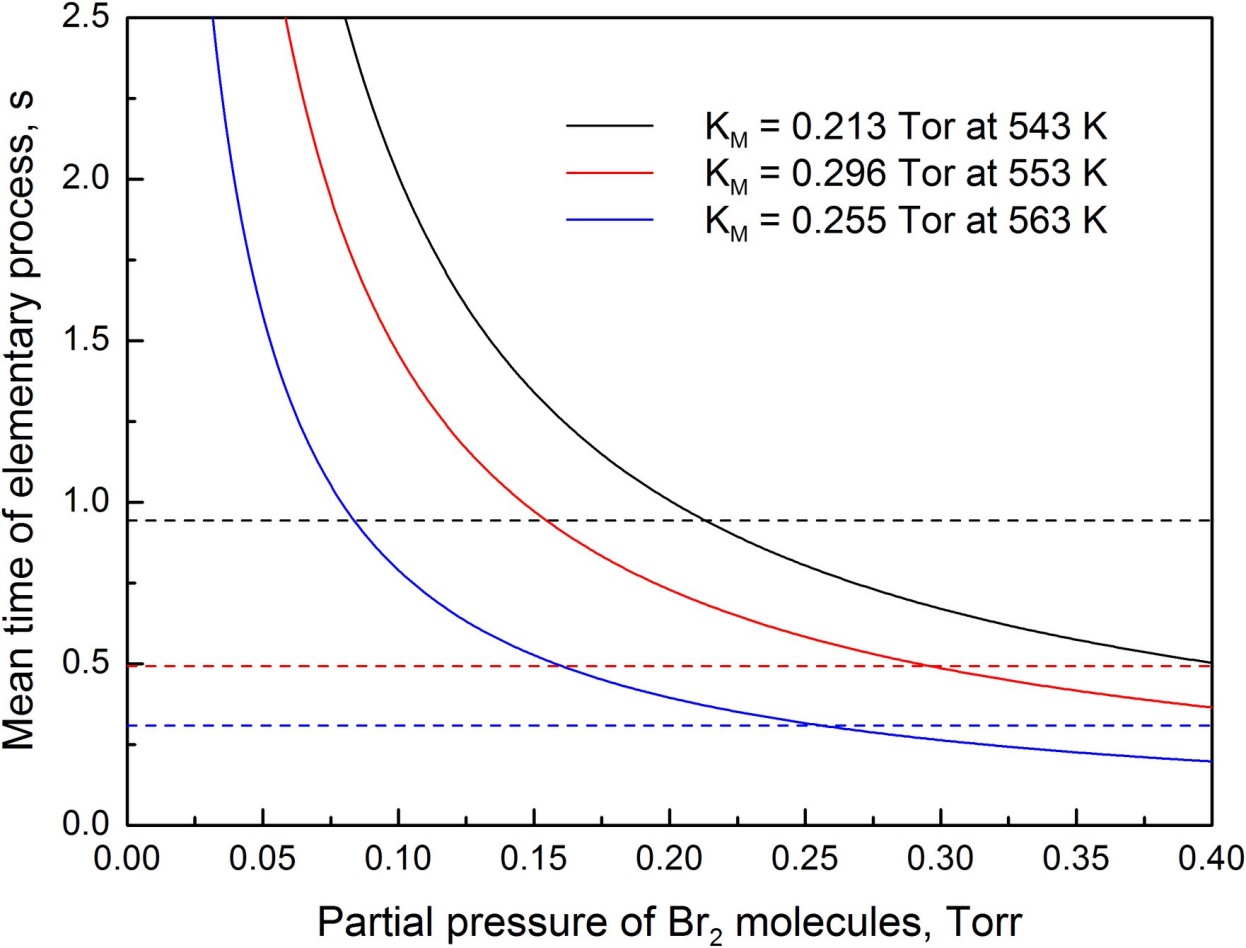
Theoretical dependences of mean times of the elementary processes on the partial pressure of Br_2_ molecules at three different temperatures. The mean desorption times of GeBr_2_ molecules are shown by the dashed lines.

The chemical etching rate of germanium substrates can also be calculated using the mean times of elementary processes. According to the model, the mean time of Ge(s)+Br_2_(g)→GeBr_2_(a) reaction is equal to τ_r_ = (k_r_p)^-1^, and the mean desorption time of GeBr_2_ molecules is equal to τ_d_ = ω^-1^. The dependences of mean times of elementary processes on the partial pressure of Br_2_ molecules at different temperatures are presented in Fig 2. It is observed that mean reaction time reciprocally decreases with the increase in partial pressure of Br_2_ molecules, while mean desorption time does not depend on the partial pressure of Br_2_ molecules. At pressure defined by the Michaelis constant, the mean reaction time becomes equal to the mean desorption time. Therefore, it is possible to state that at partial pressure p < K_M_, the etching-rate limiting process is the formation of GeBr_2_ in the adsorbed layer. While at partial pressure p > K_M_, the etching-rate limiting process is the desorption of formed GeBr_2_ molecules. The etching-rate limiting process changes when the etching rate reaches half of its maximum value.

Let us consider the etching process using another statistical approach. The reaction constant shows how many Ge atoms are removed from the surface by single Br_2_ molecule

$$\varepsilon =\frac{\Phi \left(GeBr_{2}\right)}{\Phi \left(Br_{2}\right)}=\frac{k_{r}\omega C}{k_{r}p+\omega }\sqrt{2\pi mkT},$$ (17)

where Φ(Br_2_) = p(2πmkT)^-1/2^ is the flux of Br_2_ molecules to the germanium surface, m is the mass of Br_2_ molecule, and Φ(GeBr_2_) = ω[GeBr_2_] is the flux of desorbing GeBr_2_ molecules. It is important to note that reaction constant depends on the partial pressure of Br_2_ molecules. At extremely low pressure, the reaction constant reaches its maximum value

$$\varepsilon_{0}=k_{r}C\sqrt{2\pi mkT}.$$ (18)

According to Eqs. (9) and (10), the ratio ε/ε_0_ is equal to the surface fraction not covered with adsorbate

$$\frac{\varepsilon }{\varepsilon_{0}}=\frac{\omega }{k_{r}p+\omega }=\beta .$$ (19)

**Fig 3:**
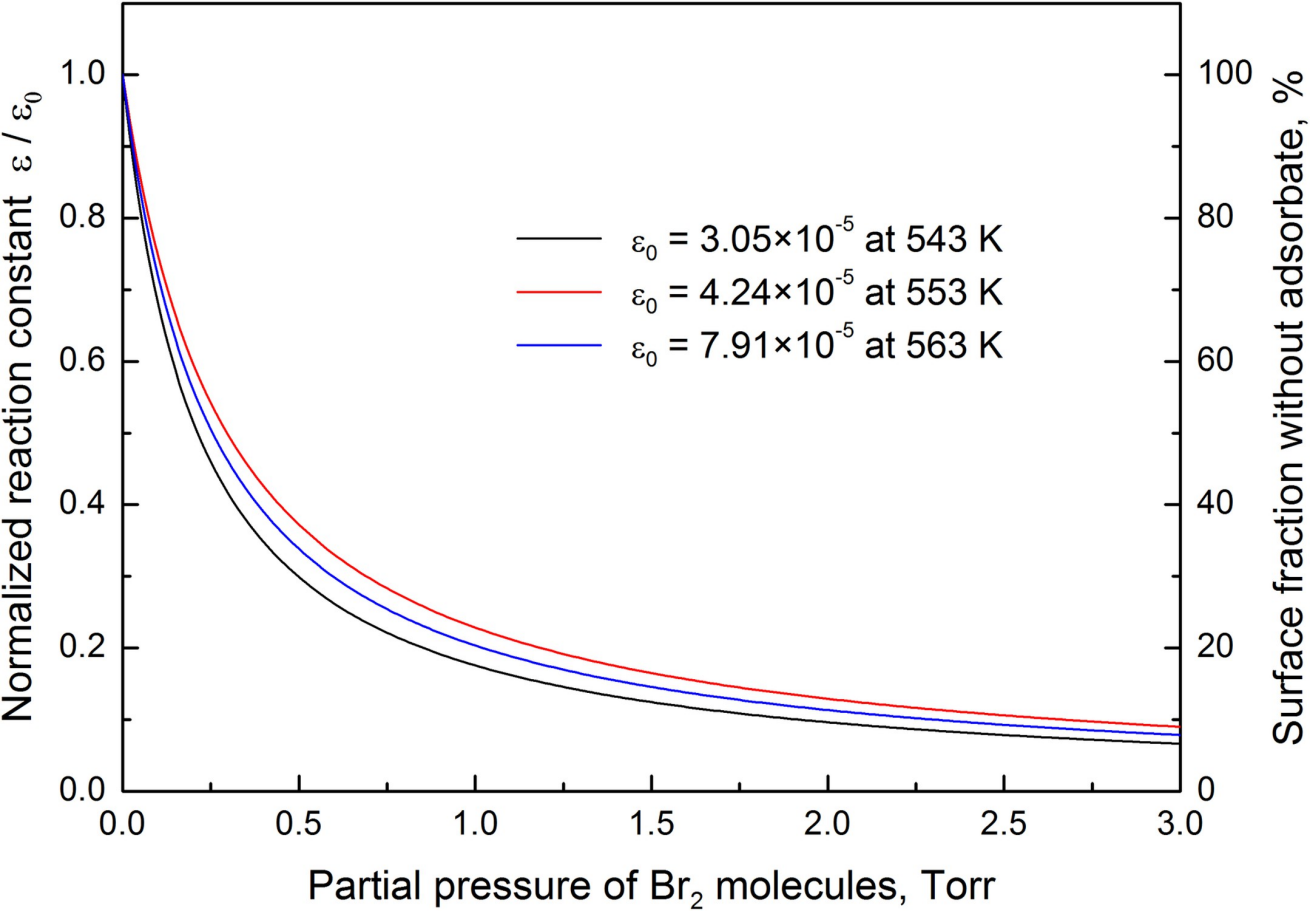
The theoretical dependences of normalized reaction constant and surface fraction not covered by adsorbate on the partial pressure of Br_2_ molecules at three different temperatures. The dependences are calculated using the rate constants of elementary processes obtained from nonlinear regression of the experimental data.

The dependences of normalized reaction constant and surface fraction not covered by adsorbate on the partial pressure of Br_2_ molecules are shown in Fig 3. The atomically clean Ge surface creates ideal conditions for the ongoing heterogeneous chemical reaction, and the normalized reaction constant reaches its highest value. With the increase in partial pressure of Br_2_ molecules, the normalized reaction constant rapidly decreases due to the accumulation of GeBr_2_ molecules in the adsorbed layer. It is important to note that the reaction product starts to accumulate in the adsorbed layer because the desorption activation energy of GeBr_2_ molecules is higher than the activation energy of Ge(s) + Br_2_(g)→GeBr_2_(a) reaction. Despite that steady-state etching rate significantly increases because of the decreased mean reaction time. The observed trend continues until partial pressure of Br_2_ molecules reaches K_M_ value. With further increase in partial pressure of Br_2_ molecules, normalized reaction constant and surface fraction not covered by adsorbate start to approach zero. The theoretical dependences derived from the experimental measurements addresses two uncertainties associated with the etching process:

1. at extremely high pressure, the surface coverage by the reaction product should suppress the etching rate because Br_2_ molecules from the gas phase cannot chemisorb on the surface. Theoretical calculations indicate that lowest value of the normalized reaction constant is achieved at temperature T = 543 K. At partial pressure 200 Torr, the ratio is equal to ε/ε_0_ = 1.064×10^−3^. It yields the reaction constant ε = 3.24×10^−8^, which indicates that 99.9999967% of incident Br_2_ molecules are reflected from the substrate surface. Under the considered conditions, single Br_2_ molecule chemisorbs after 30 million collisions with the Ge surface. Other molecules are scattered from the surface back to the gas phase;
2. the maximum etching rate does not depend on the partial pressure of Br_2_ molecules. This uncertainty can be addressed only experimentally. The experimental measurements [12] confirm that chemical etching rate is not suppressed at high partial pressure of Br_2_ molecules due the increased surface coverage by the reaction product. This also means that intermediate reaction product does not accumulate on the Ge surface. During the etching process GeBr radicals are rapidly converted to GeBr_2_ molecules, which subsequently desorb. The considered mechanism eradicates possibility of the surface passivation, and the maximum etching rate at high partial pressure of Br_2_ molecules remains unchanged.

**Fig 4:**
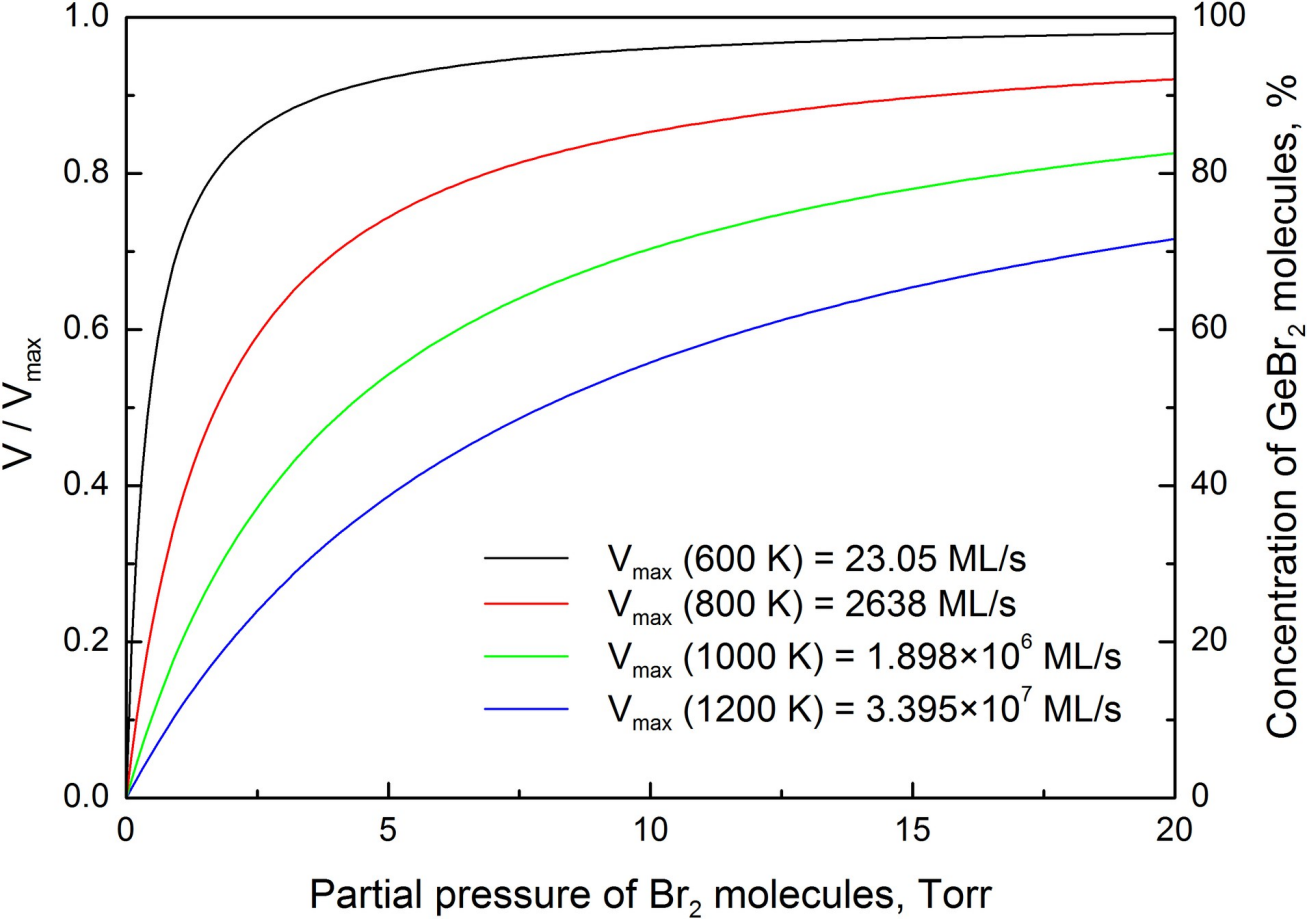
The theoretical dependences of V/V_max_ and concentration of GeBr_2_ molecules in the adsorbed layer on the partial pressure of Br_2_ molecules at four different temperatures.

The etching rate is proportional to the concentration of GeBr_2_ molecules in the adsorbed layer. However, the dependence of surface coverage by the reaction product on the temperature is not pronounced in Fig 3. At constant partial pressure, the surface coverage is lowest at 553 K and highest at 543 K. Meanwhile, the intermediate value of the surface coverage by GeBr_2_ molecules is achieved at temperature 563 K. The theoretical dependences of normalized reaction constant and surface fraction without adsorbate on the partial pressure of Br_2_ molecules are affected by the fitting errors. This means that during the experiment [12] the etching rate was not measured precisely enough in the considered temperature range. Let us to calculate the chemical etching rate of germanium substrates at higher temperatures using the derived activation energies of the elementary processes. The theoretical dependences of V/V_max_ on the partial pressure of Br_2_ molecules at different temperatures are shown in Fig 4. According to Eqs. (10) and (13), the ratio V/V_max_ is equal the concentration of GeBr_2_ molecules in the adsorbed layer. It is observed that at constant partial pressure, the concentration of GeBr_2_ molecules in the adsorbed layer decreases with the increase in temperature. As a result, V_max_/2 is achieved at higher partial pressure of Br_2_ molecules. This indicates that Michaelis constant depends on temperature.

Let us investigate the relationship between dry etching processes and Michaelis–Menten kinetics more closely. Michaelis–Menten kinetics is based on the following reaction scheme:

$$\text{E}+\text{S}\overset{{\text{k}}_{\text{fwd}}}{\underset{{\text{k}}_{\text{rev}}}{⇌}}\text{ES}\overset{{\text{k}}_{\text{cat}}}{\to }\text{E}+\text{P},$$ (20)

where E is the enzyme, ES is the intermediate compound, S is the substrate, and P is the product. The Michaelis constant is equal to

$$K_{M}=\frac{k_{rev}+k_{cat}}{k_{fwd}},$$ (21)

where k_cat_ is the rate constant of the catalytic reaction, k_fwd_ is the rate constant of the intermediate compound formation, and k_rev_ is the rate constant of the reversible reaction. In the case of dry etching processes, $k_{cat}\equiv \omega$ and $k_{fwd}\equiv k_{r}$. This means that desorption of GeBr_2_ molecules is the elementary process responsible for the increased etching rate. During chemical etching of silicon with halogen molecules, the escape of bystanding Si atom from the reaction zone not only stabilizes the reaction product but also reduces desorption activation energy of the formed silicon dihalide molecule. This type of catalysis was predicted theoretically [19] and confirmed experimentally [20]. It is highly likely that dry etching of germanium substrates is catalyzed in the same way. Both, silicon and germanium crystals have face-centered diamond-cubic structures. The reversible reaction becomes plausible when the mean desorption time of GeBr_2_ molecules exceeds the mean reaction time. The described situation occurs when the partial pressure of Br_2_ molecules exceeds pressure defined by the Michaelis constant, p > K_M_. During dry etching processes it converts reaction product into reactant. The inclusion of reversible reaction in the model yields too complex steady-state etching-rate expression, which cannot be converted into the Michaelis–Menten equation. Additionally, the described behavior cannot be attributed to single enzyme, and Michaelis constant retains the earlier introduced form:

$$K_{M}=\frac{\omega }{k_{r}}=A^{-1}\exp\left(\frac{E_{r}-E_{d}}{kT}\right).$$ (22)

**Fig 5:**
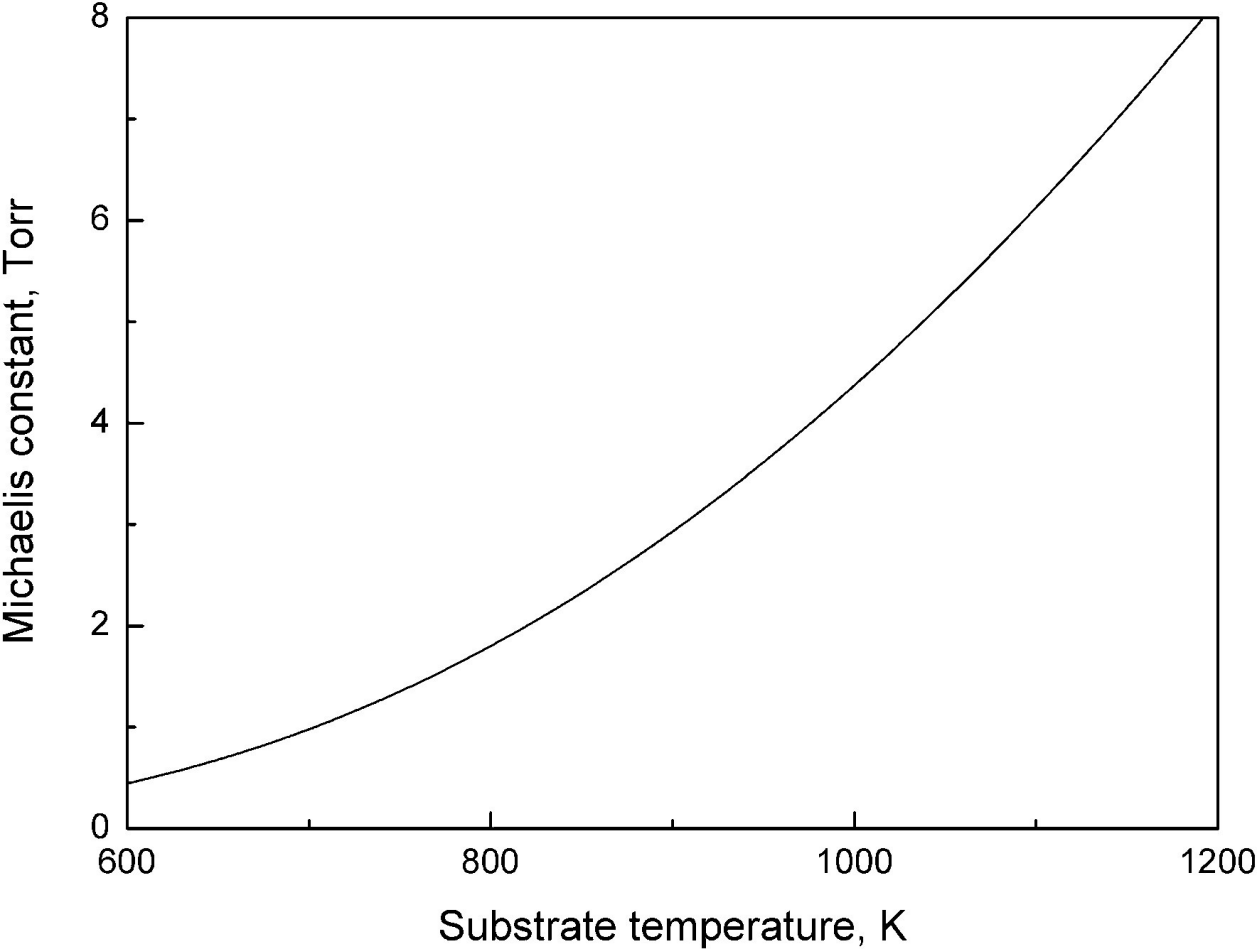
The theoretical dependence of Michaelis constant on temperature. Michaelis constant is calculated using the derived activation energies of elementary processes.

The difference between activation energy of Ge(s) + Br_2_(g)→GeBr_2_(a) reaction and desorption activation energy of GeBr_2_ molecules results in the temperature dependence of Michaelis constant. The theoretical dependence of Michaelis constant on temperature is shown in Fig 5. The Michaelis constant increases more than 20 times when temperature is raised from 550 K to nearly melting point. The experimental measurements confirm that for certain enzyme-catalyzed reactions Michaelis constant depends on temperature [21–24].

The Michaelis–Menten equation successfully describes the chemical etching rate of other materials when, in the certain range of partial pressure, conditions required for the Michaelis–Menten kinetics are fulfilled. The most important experimental observations of the Michaelis–Menten kinetics during dry etching processes are following:

1. chemical etching of SiO_2_ films in the fluorine-based plasmas [25];
2. chemical etching of SiGe alloys using xenon difluoride vapor [26];
3. chemical etching of silicon substrates in the fluorine-based plasma at cryogenic and room temperatures [27];
4. atomic layer etching of Al_2_O_3_ films using the sequential exposures to HF and trimethylaluminum [28].

The numerous experiments extend validity of the Michaelis–Menten kinetics for the inorganic materials over wide temperature range, and provide insights into the processes taking place at the atomic scale.

## 5. Conclusions

The relationship between dry etching processes and enzyme-catalyzed chemical reactions is established. The chemical etching of germanium in Br_2_ environment at elevated temperatures is described by the Michaelis–Menten equation. Reaction rate constants and desorption rate constants are obtained using nonlinear regression of the experimental data. Subsequently, the activation energies of elementary processes are evaluated using TST. It is found that the activation energy of Ge(s) + Br_2_(g)→GeBr_2_(a) reaction is equal to (1.168 ± 0.173) eV, and the desorption activation energy of GeBr_2_ molecules is equal to (1.397 ± 0.014) eV. The difference between reaction activation energy and desorption activation energy results in the temperature dependence of Michaelis constant.
